# Exploring the Impact of Beta-Blockers Post-Acute Myocardial Infarction in Patients with Preserved Ejection Fraction: A Meta-Analysis

**DOI:** 10.3390/jcm14113969

**Published:** 2025-06-04

**Authors:** Khalid A. Alnemer

**Affiliations:** Department of Internal Medicine, College of Medicine, Imam Mohammad Ibn Saud Islamic University (IMSIU), Riyadh 13317, Saudi Arabia; aalnemerkhalid@gmail.com

**Keywords:** beta-blockers, preserved, acute myocardial infarction, left ventricular ejection fraction

## Abstract

**Background/Objectives:** Previous research has established that beta-blockers significantly reduce all-cause mortality, cardiovascular mortality, and recurrent acute myocardial infarction (AMI) in patients with left ventricular dysfunction following AMI. However, their efficacy in patients with preserved left ventricular ejection fraction (LVEF) who undergo timely reperfusion and revascularization while receiving evidence-based medical management remains inconclusive. To address this uncertainty, we conducted a systematic review and meta-analysis to synthesize the available evidence on the impact of beta-blocker therapy in patients with AMI and preserved LVEF. **Methods:** A comprehensive literature search was conducted across PubMed, the Web of Science, and Scopus from their inception until November 2024. The search strategy incorporated three primary keywords and their corresponding Medical Subject Headings (MeSH) terms: “preserved”, “myocardial infarction”, and “beta-blocker”. Data analysis was performed using Review Manager 5.4 software. A random-effects model was applied to account for the study’s heterogeneity, while a fixed-effects model was utilized for homogeneous outcomes. Pooled odds ratios (ORs) and hazard ratios (HRs) were calculated for dichotomous outcomes, with a 95% confidence interval (CI) and a significance threshold of *p* < 0.05. **Results:** Beta-blocker therapy was significantly associated with a reduction in all-cause mortality compared to non-use, with an OR of 0.73 (95% CI: 0.61–0.88, *p* = 0.001) and an HR of 0.78 (95% CI: 0.67–0.91, *p* = 0.002). Similarly, beta-blocker administration was linked to a lower risk of cardiovascular mortality, demonstrating an OR of 0.76 (95% CI: 0.68–0.84, *p* < 0.00001) and an HR of 0.76 (95% CI: 0.59–0.99, *p* = 0.04). Furthermore, beta-blocker use was significantly correlated with a decreased risk of major adverse cardiovascular events (MACEs) compared to non-use, with an OR of 0.84 (95% CI: 0.75–0.95, *p* = 0.004) and an HR of 0.84 (95% CI: 0.71–0.99, *p* = 0.04). **Conclusions:** The current meta-analysis suggests a potential beneficial association between beta-blocker use and outcomes in patients with AMI and preserved LVEF, including lower rates of all-cause mortality, cardiovascular mortality, and MACEs; however, these findings should be interpreted with caution due to the observational nature of most included studies. Therefore, further randomized controlled trials (RCTs) are needed to confirm these findings, particularly in distinguishing outcomes among patients with and without heart failure.

## 1. Introduction

In recent years, primary percutaneous coronary intervention (PCI) has emerged as the most common therapy for patients with acute myocardial infarction (AMI). Prompt reperfusion of the obstructed coronary artery in AMI patients enhances clinical outcomes and left ventricular ejection fraction (LVEF) [1]. Medical interventions utilizing renin–angiotensin system (RAS) inhibitors, beta-blockers, and statins have enhanced therapeutic results [2,3,4]. Before the implementation of reperfusion therapy in clinical practice, beta-blockers were often utilized in patients with AMI. Beta-blockers function as antiarrhythmic drugs and diminish myocardial expenditure of oxygen, hence expediting myocardial necrosis by regulating heart rate and myocardial contraction during the acute phase of AMI [5]. Beta-blockers confer a survival advantage and inhibit left ventricular remodeling in patients exhibiting left ventricular dysfunction following acute myocardial infarction [3,6]. Consequently, numerous guidelines advocate for the administration of beta-blockers in patients exhibiting reduced left ventricular ejection fraction (LVEF) or heart failure following AMI, including both ST-segment elevation myocardial infarction (STEMI) and non-STEMI (class I) [7,8,9].

The effectiveness of beta-blockers in individuals with heart failure and diminished ejection fraction is widely established. Clinical trials have demonstrated that prolonged beta-blocker medication following MI decreases mortality by roughly 20% [10,11,12]. Nonetheless, these findings originate from trials predominantly comprising individuals with extensive MI and left ventricular systolic dysfunction, undertaken primarily in the 1980s. This period precedes developments including high-sensitivity cardiac troponins, PCIs, antithrombotic medications, high-intensity statins, and renin–angiotensin–aldosterone system antagonists. A meta-analysis indicated that in the context of contemporary reperfusion methods, beta-blockers did not markedly decrease fatality rates [13]. Contemporary, well-powered randomized controlled trials (RCTs) provide scant data on the impact of long-term beta-blocker medication in individuals with AMI and preserved LVEF [14,15].

Previous research demonstrates that in instances of left ventricular dysfunction after acute myocardial infarction (AMI), beta-blockers reduce the occurrence of all-cause mortality, cardiovascular mortality, and recurrent AMI [3]. Nonetheless, the effectiveness of beta-blockers in patients with maintained left ventricular ejection fraction (LVEF) undergoing rapid perfusion and revascularization while receiving medical care based on evidence such as potent dual antiplatelet therapy, high-dose angiotensin-converting enzyme inhibitors (ACEis), angiotensin II receptor blockers (ARBs), and effective statins remains mostly unresolved [16]. To address this debate, we performed a systematic review and meta-analysis to aggregate the findings from all existing studies examining the use of beta-blockers following AMI in patients with preserved LVEF.

## 2. Materials and Methods

This meta-analysis was conducted based on the PRISMA guidelines [17] by searching for all eligible articles on PubMed, the Web of Science, and Scopus from inception till November 2024. A search strategy depending on three main keywords and utilizing their Medical Subject Headings (MESH) terms: “preserved” AND “myocardial infarction” AND “beta-blocker”. We used the title and abstract filter on PubMed and the abstract filter on Scopus and the Web of Science to narrow the scope and specify the results (Supplementary Table S1).

***Screening-based criteria***

After the searching process, the studies were loaded into Rayyan to streamline the screening process steps. The initial screening utilized titles and abstracts, followed by a comprehensive full-text review to assess the consideration of the studies to be included in the current meta-analysis. The study involved a population of patients with AMI and preserved LVEF (≥50% but other studies reported it as ≥40%, especially in populations with broader inclusion criteria, such as those with higher-risk patients or those with early-stage heart failure), utilizing beta-blockers as the intervention, with no beta-blocker use as the comparator, and assessing outcomes including mortality, major adverse cardiovascular events (MACEs), recurrent MI, and rehospitalization. The research design specifications prioritized observational studies (cohort and case-control) and RCTs. Case reports and reviews were excluded.

***Data extraction***

The fundamental characteristics of the investigations, encompassing research design, gender, age, sample size, LVEF, and previous PCI, were gathered utilizing Microsoft Excel spreadsheets. Outcome data, encompassing all-cause mortality, cardiac mortality, MACEs, recurrent MI, and rehospitalization, together with hazard ratios (HRs), event counts, and total numbers, were also collected.

***Quality assessment***

Diverse assessment tools were used in accordance with the study design. The Newcastle–Ottawa Scale (NOS), which assigns a star rating from 0 to 9 for each study, was utilized to assess the quality of observational studies [18]. For RCTs, the Cochrane risk-of-bias tool (Rob-2), which includes five dimensions, was used [19].

***Statistical analysis***

The meta-analysis was conducted using Review Manager 5.4 software. The analysis employed a random-effects model to address the intrinsic heterogeneity across the studies and a fixed-effect model for homogeneous outcomes, utilizing a 95% confidence interval (CI) and a significance level (*p*-value) of 0.05. The pooled odds ratio (OR) was calculated for dichotomous variables, in addition to the pooled HR. The heterogeneity was assessed utilizing I2 and a *p*-value of 0.05. Sensitivity analysis using subgrouping based on study designs was conducted to resolve the heterogeneity, and funnel plots were used to assess the publication bias.

## 3. Results

**Figure 1:**
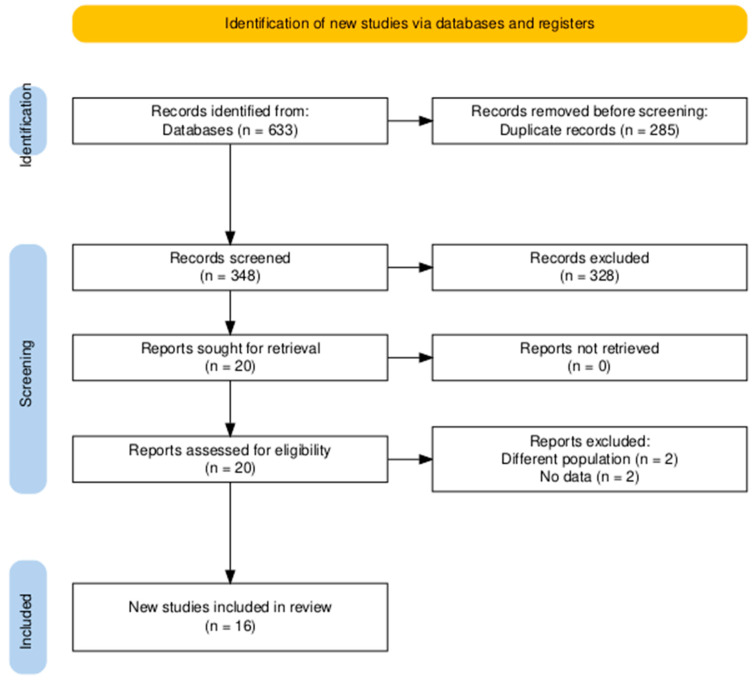
PRISMA flow diagram of searching and screening processes.

The executed search method produced 633 entries, of which 285 were identified as duplicates. Upon assessing the titles and abstracts of the remaining 348 papers, 20 met the criteria for a comprehensive review of the full text. In conclusion, 16 publications were considered appropriate for inclusion in the final meta-analysis [14,15,20,21,22,23,24,25,26,27,28,29,30,31,32,33] (Figure 1).

***Baseline characteristics***

The current systematic review and meta-analysis included 16 articles encompassing 3 RCTs and 13 cohort studies comparing the use of beta-blockers against no use in patients with MI and preserved ejection fraction. The studies included a total of 57,901 patients who took beta-blockers and 20,654 who did not take them. Some patients had a history of PCI, and others did not. Follow-up ranged from 1 to 62 months. Baseline characteristics of the included patients are shown in Table 1.

***Quality and risk of bias assessment***

**Figure 2:**
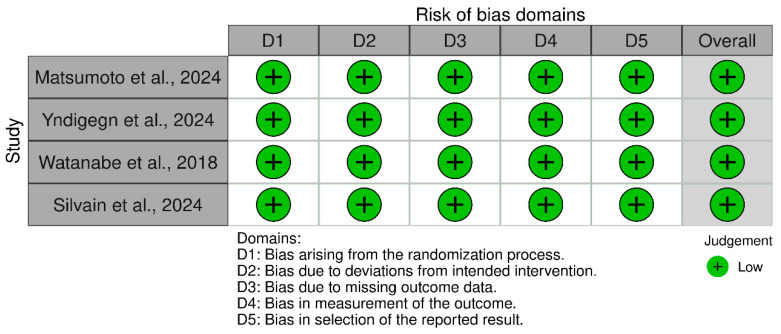
Risk of bias assessment of RCTs using Rob-2 [14,15,31,33].

The 12 included cohort studies assessed using NOS were found to be of high quality (Table 2). Also, the three RCTs assessed using Rob-2 were deemed to be of low risk of bias (Figure 2).

***Meta-analysis***

**Figure 3:**
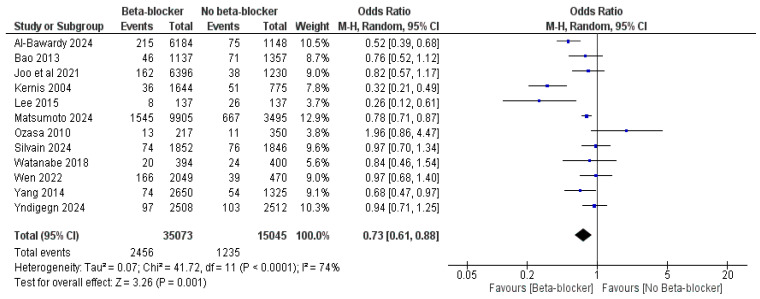
The effect of beta-blockers on the risk of all-cause mortality using the odds ratio.

**Figure 4:**
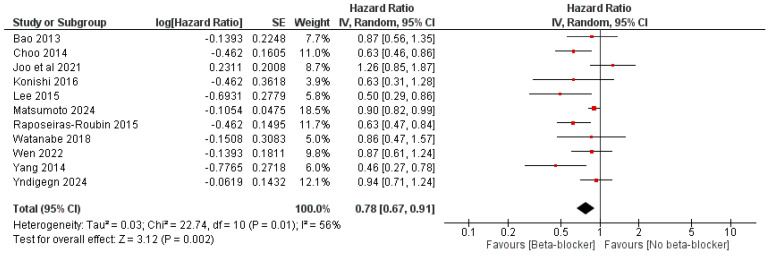
The effect of beta-blockers on the risk of all-cause mortality using the hazard ratio.

The use of beta-blockers was significantly associated with reduced all-cause mortality compared with no beta-blocker use, with an OR of 0.73 (95% CI: 0.61, 0.88, *p* = 0.001) and I^2^ = 74%, *p* < 0.0001, and an HR of 0.78 (95% CI: 0.67, 0.91, *p* = 0.002) and I^2^ = 56%, *p* = 0.01 (Figure 3 and Figure 4).

**Figure 5:**
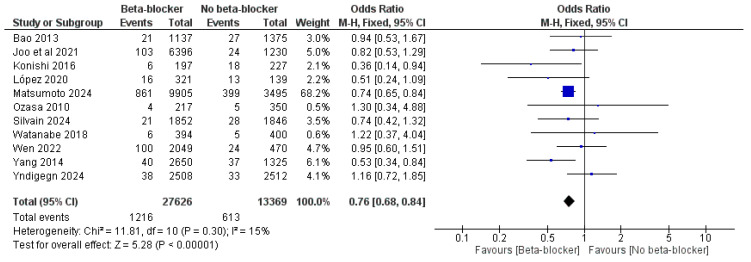
The effect of beta-blockers on the risk of cardiac mortality using the odds ratio.

**Figure 6:**
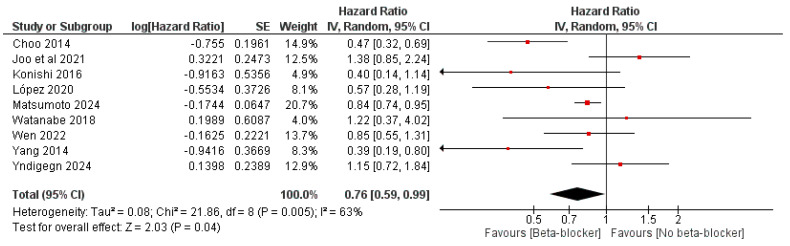
The effect of beta-blockers on the risk of cardiac mortality using the hazard ratio.

Also, the use of beta-blockers led to decreased risk of cardiac death, with an OR of 0.76 (95% CI: 0.68, 0.84, *p* < 0.00001) and I^2^ = 15%, *p* = 0.3, and an HR of 0.76 (95% CI: 0.59, 0.99, *p* = 0.04) and I^2^ = 63%, *p* = 0.005 (Figure 5 and Figure 6).

**Figure 7:**
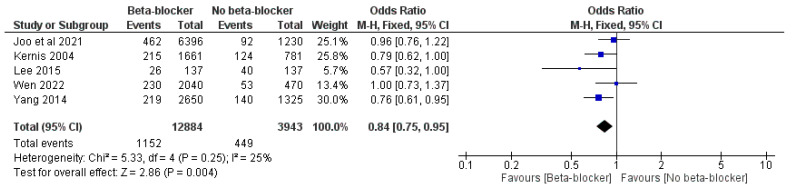
The effect of beta-blockers on the risk of MACEs using the odds ratio.

**Figure 8:**
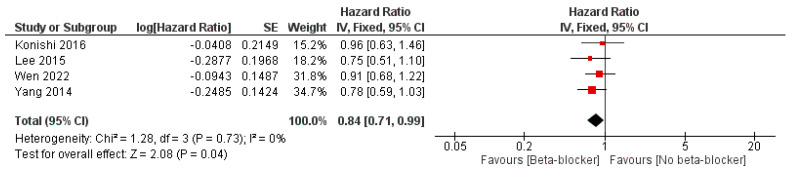
The effect of beta-blockers on the risk of MACE using the hazard ratio.

Moreover, the use of beta-blockers was significantly associated with reduced risk of MACEs compared with no beta-blockers, with an OR of 0.84 (95% CI: 0.75, 0.95, *p* = 0.004) and I^2^ = 25%, *p* = 0.25, and an HR of 0.84 (95% CI: 0.71, 0.99, *p* = 0.04) and I^2^ = 0% (Figure 7 and Figure 8).

On the other hand, no difference was observed between beta-blockers and non-beta-blockers with regard to recurrent MI, with an OR of 0.97 (95% CI: 0.83, 1.14, *p* = 0.73) and an HR of 0.98 (95% CI: 0.82, 1.17, *p* = 0.79). Also, no difference was observed between both groups regarding rehospitalization, with an OR of 0.94 (95% CI: 0.8, 1.11, *p* = 0.89) and an HR of 0.91 (95% CI: 0.65, 1.27, *p* = 0.57; Supplementary Figures S1–S4).

*Subgroup analysis and publication bias*

The subgroup analysis of all-cause mortality according to study designs (RCTs and cohort studies) showed the resolution of heterogeneity in the RCT subgroup, with the persistence of heterogeneity in the cohort studies (I^2^ = 78%, *p* < 0.0001). Also, in the subgroup analysis of cardiac death, the RCT subgroup showed a narrow CI with the absence of heterogeneity, while the cohort subgroup showed a broad CI with the presence of heterogeneity. However, the results in the RCT subgroups were insignificant, which can be explained by the small sample size that failed to reach statistical power (Supplementary Figures S5 and S6).

The funnel plot of all-cause mortality showed the presence of risk of publication bias, while that of cardiac mortality showed minimal risk of publication bias based on the OR, while the funnel plots of HR showed minimal risk of bias in all-cause mortality and the presence of publication bias in cardiac mortality (Supplementary Figures S7–S10).

## 4. Discussion

This meta-analysis examined the effects of beta-blockers in AMI patients with intact ejection fractions, regardless of heart failure status. The aggregation of results utilizing the OR and HR indicated that the administration of beta-blockers was more effective than non-administration in decreasing the risk of all-cause and cardiac mortality, as well as in mitigating the risk of MACEs. Nonetheless, they were analogous in their impact on recurrent myocardial infarction and readmission.

A 2014 meta-analysis demonstrated that beta-blockers provided neither mortality reduction nor net benefit in myocardial infarction patients without heart failure during the reperfusion era, unlike the pre-reperfusion era [13]. Although there has been a decline in MI and angina throughout the reperfusion era, this has coincided with a rise in heart failure and cardiogenic shock, along with an elevated rate of drug cessation [13]. Although the exclusion criteria of this meta-analysis included post-MI heart failure trials, not all included trials only studied individuals with preserved LVEF. Primarily, they were predominantly outdated trials, with only two published subsequent to 2005 [13].

A later meta-analysis published in 2015 demonstrated that all-cause and cardiac mortality were lower in the beta-blocker treatment group compared to the non-beta-blocker group, although this finding was confined to individuals with decreased LVEF. Nonetheless, they incorporated observational research, some of which predates the reperfusion period [34].

A recent meta-analysis published in 2019, which included all 16 trials from the 2000s, demonstrated a 26% decrease in all-cause mortality linked to beta-blocker therapy after MI without heart failure. However, that effect dissipated when considering bias. The vast majority of the studies analyzed did not establish a pre-defined LVEF, with only four specifying an LVEF beyond 40% (two of which had an LVEF of 50% or higher) [35].

PCI has undergone notable changes over the years, influenced by advancements in medical technology, evolving clinical practices, and insights from RCTs. An analysis of the British Cardiovascular Intervention Society (BCIS) registry, encompassing data from 1,245,802 PCI procedures in England and Wales between 2006 and 2019, revealed that elective PCI rates per 100,000 population increased from 50.7 in 2006 to 58.4 in 2019 [36]. Furthermore, a systematic review of 25 all-comers trials involving 66,327 patients highlighted improvements in PCI-related outcomes over time. The study observed a decrease in the incidence of target lesion revascularization and stent thrombosis, potentially attributed to advancements in PCI technologies, techniques, and adjunctive medical therapies [37].

Numerous registries have been published that corroborate the absence of a protective effect from beta-blockers. Nonetheless, several constraints encompass the absence of a pre-established LVEF. A substantial registry dataset from the UK, comprising over 170,000 patients hospitalized with AMI from 2007 to 2013, indicated an overall one-year mortality rate of 5.2% [38]. Although the unadjusted 1-year death rate was lower in the beta-blocker cohort than in the non-beta-blocker cohort (4.9% vs. 11.2%; *p* < 0.001), the adjusted mortality rates were similar between the two groups. A limitation of the research they conducted is that the definition of heart failure was based on hospital records, which did not uniformly include a pre-defined LVEF and depended on a clinical diagnosis of heart failure [38].

Following a STEMI incident, beta-blocker medication may confer its advantageous effects by mitigating excessive stimulation of cardiovascular beta-adrenergic receptors, induced by elevated catecholamine levels [39]. This method decreases intracellular levels of cyclic adenosine monophosphate and calcium, resulting in diminished cardiac contractility, systemic arterial pressure, and heart rate, which are critical factors in myocardial oxygen consumption [40]. The decrease in myocardial oxygen demand underpins its anti-ischemic efficacy in myocardial regions at risk due to compromised coronary flow [41]. Moreover, lowering the heart rate and diminishing blood pressure improves left ventricular flexibility. The prolongation of diastole improves perfusion of the ischemic myocardium, particularly in the subendocardial region, hence diminishing infarct size [42,43]. Furthermore, beta-blockers may diminish the elevation in endothelial shear, platelet aggregation, and blood viscosity, hence decreasing the likelihood of new coronary plaque rupture or thrombosis [44,45]. Beta-blockers are associated with a diminished risk of ventricular fibrillation and fatal cardiac arrest via processes that may include the extension of the effective ventricular refractory period, the inhibition of triggered activities and automaticity, and a decrease in electrophysiological diversity [46].

During the pre-fibrinolytic phase, the treatment of beta-blockers during and following a STEMI event was significantly correlated with reduced mortality, cardiac arrest, and reinfarction rates [10,12]. The introduction of fibrinolysis and antiplatelet therapy as primary treatments for STEMI resulted in the diminished efficacy of beta-blockers in this context.

The current ESC guidelines for STEMI care recommend using intravenous beta-blockers upon presentation in patients undergoing primary PCI, provided there are no contraindications, along with normal oral medication throughout hospitalization and its continuation thereafter [47]. The American College of Cardiology Foundation/American Heart Association STEMI recommendations also encourage the commencement of oral beta-blockers within the first 24 h, with maintenance during hospitalization and at discharge [8].

Most of the supporting data for the mid- and long-term usage of beta-blockers originates from trials conducted in the pre-reperfusion era, as demonstrated in the systematic review by Freemantle et al. [48]. Recent evidence has revealed conflicting outcomes, indicating a significant relationship with LVEF [21,49].

Multiple credible hypotheses account for the lack of predictive benefit of beta-blockers in AMI patients without heart failure and with preserved LVEF. Research demonstrates that beta-blockers confer beneficial effects by reducing lethal arrhythmias, myocardial ischemia, and reinfarction; however, patients with preserved LVEF may display less myocardial scarring and a higher quantity of surviving cardiomyocytes when compared with AMI patients with diminished LVEF [24,50]. Secondly, sympathetic activity is heightened in the presence of AMI. Beta-blockers may impede myocardial remodeling by reducing oxygen consumption and avert arrhythmias by slowing the heart rate. Administering prompt reperfusion therapy during acute myocardial infarction may suppress sympathetic activity, with benefits similar to those of beta-blockers [38,50]. Thirdly, beta-blockers are essential in the management of heart failure with reduced ejection fraction; however, there is limited evidence supporting their use in heart failure with mid-range ejection fraction or heart failure with preserved ejection fraction [51].

Various factors are associated with worse outcomes in coronary artery disease patients. Data from 23,270 patients enrolled in the international PRAISE registry who were discharged after acute coronary syndrome were analyzed to assess the impact of in-hospital bleeding on one-year outcomes. The findings indicated that in-hospital bleeding serves as a key marker of frailty in acute coronary syndrome patients, highlighting the need for increased vigilance during follow-up for those affected [52].

Several trials are still being conducted in this field, and the results are pending, warranting future meta-analyses to include further findings [53,54,55].

The current meta-analysis provides evidence on the use of beta-blockers after AMI in patients with preserved ejection fraction. However, some limitations are still present, including the small number of RCTs and the majority of cohort studies that act as a source of bias. The considerable heterogeneity observed in the meta-analysis (I^2^ = 74% for all-cause mortality) may stem from several factors. Differences in study populations, such as variations in age, gender distribution, comorbidities, follow-up periods, and geographic healthcare settings, could contribute to variability in outcomes. Additionally, methodological differences, including study design (randomized controlled trials vs. observational studies), intervention protocols, and follow-up durations, may introduce inconsistencies. Also, there are different characteristics of the patients, as some of them underwent PCI, and others did not; some of them take specific medications, some of them have heart failure, and others do not. Among the limitations is the non-previous registration of the study. Without an a priori statistical analysis plan, there is a risk that statistical methods could be chosen based on the data rather than pre-defined criteria, but this was not done, as we reported the results as the analysis indicated to overcome this risk. A limitation of this meta-analysis is the variability in the definition of preserved LVEF across the included studies. While LVEF ≥ 50% is commonly used as the threshold for preserved ejection fraction, some studies may have used different cut-off points (e.g., ≥45% or ≥40%), which could affect the generalizability of the results; however, this was minimally present, and we included all the studies mentioning the word “preserved”. The asymmetry observed in the funnel plots for all-cause and cardiac mortality suggests a risk of publication bias. This is a critical limitation, as studies with non-significant or negative results may remain unpublished, leading to an over-representation of studies demonstrating a beneficial effect of beta-blockers. Therefore, while the findings of this meta-analysis suggest a potentially protective effect of beta-blockers in AMI patients with preserved LVEF, they must be interpreted cautiously. Future research should prioritize the inclusion of unpublished data and results from ongoing trials to reduce the impact of publication bias. Additionally, registering protocols prospectively and encouraging publication regardless of study outcome are essential steps to enhance evidence transparency and reduce bias. We recommend the specification of future RCTs that should be conducted to assess the effectiveness of beta-blockers in AMI patients with preserved LVEF and having heart failure, as well as without heart failure.

## 5. Conclusions

The pooling of the included studies showed the efficacy of beta-blockers in AMI patients with preserved LVEF in lowering the risk of all-cause mortality and cardiac mortality in addition to a decreased risk of MACEs compared with patients who did not receive beta-blockers. However, future RCTs focusing on heart failure and non-heart failure are still warranted to validate the present findings.

## Figures and Tables

**Table 1:** Baseline characteristics of the included patients.

Study ID	Design	Sample Size		Age		Male, n (%)		LVEF, Mean (SD)		Previous PCI		Follow-Up, Months
		Beta-Blocker	No Beta-Blocker	Beta-Blocker	No Beta-Blocker	Beta-Blocker	No Beta-Blocker	Beta-Blocker	No Beta-Blocker	Beta-Blocker	No Beta-Blocker	
Ozasa et al., 2010 [29]	Cohort	349	561	66.4 (11.5)	68 (11.6)	264 (75)	430 (76)	51 (11.9)	53.2 (12.2)	41 (11)	79 (14)	12
López et al., 2020 [28]	Cohort	417	43	63.8 (13.8)		317 (76)	31 (70.1)	NR	NR	396 (95)	36 (90.7)	47.7
Al-Bawardy et al., 2024 [20]	Cohort	15,541	2798	55.3 (12.1)	27.4 (13.5)	12,510 (80.5)	2029 (72.5)	NR	NR	1756 (22.2)	154 (12.4)	1–12
Choo et al., 2014 [27]	Cohort	2424	595	60.9 (12.1)	63.1 (12.8)	1799 (74.2)	412 (69.2)	NR	NR	NR	NR	1–12
Matsumoto et al., 2024 [31]	Cohort	9905	3495	72.1 (8.4)	43.6 (8.5)	4510 (45.5)	1512 (43.3)	59.4 (7.1)	61 (7.8)	1681 (21.6)	280 (14)	34.1
Kernis et al., 2004 [26]	Cohort	1661	781	60 (12)	62 (12)	1240 (75)	560 (72)	49.2 (12)	48.1 (13)	146 (8.8)	98 (13)	6
Lee et al., 2015 [25]	Cohort	589	303	56 (12)	61 (13)	491 (82.1)	225 (74.3)	53 (10)	49 (12)	40 (6.7)	25 (8.3)	54
Yndigegn et al., 2024 [14]	RCT	2508	2512	65 (2.3)	65 (2.3)	1945 (77.6)	1944 (77.4)	NR	NR	147 (5.9)	175 (7)	13
Watanabe et al., 2018 [15]	RCT	394	400	63.9 (11.2)	64.5 (11.3)	327 (83)	312 (78)	58.1 (8.6)	58 (8.9)	20 (5.1)	21 (5.3)	12
Joo et al., 2021 [24]	Cohort	10,251	1949	63.2 (12.5)	65.6 (12.9)	7655 (74.7)	1414 (72.4)	52.2 (10.8)	52.5 (12.1)	NR	NR	24
Konishi et al., 2016 [23]	Cohort	197	227	64 (12.1)	64.2 (11.1)	152 (77.2)	184 (81.1)	55.6 (9.5)	57.4 (9.9)	NR	NR	6
Wen et al., 2022 [32]	Cohort	2049	470	62 (2.6)	64 (3)	1624 (79.5)	101 (21.5)	58 (1.02)	60 (1.2)	1673 (81.6)	331 (70.4)	1
Bao et al., 2013 [22]	Cohort	1614	2078	65.8 (12.2)	68 (12.1)	1255 (77.8)	1500 (72.2)	52.4 (12.6)	54.3 (12.2)	NR	NR	3
Raposeiras-Roubin et al., 2015 [30]	Cohort	2277	959	63.8 (12)	67.8 (11.5)	1692 (74.3)	407 (68.4)	NR	NR	1667 (73.2)	340 (57.1)	62
Yang et al., 2014 [21]	Cohort	6873	1637	62 (2.7)	65 (2.8)	5182 (75.4)	1217 (74.3)	51 (2)	50 (2.5)	285 (4.1)	83 (5.1)	12–24
Silvain et al., 2024 [33]	RCT	1852	1846	63.5 (10.9)	63.5 (11.2)	1531 Silvain et al., 2024 (82.7)	1530 (82.9)	57.2 (5.9)	57.2 (5.9)	1693 (91.4)	1709 (92.5)	6

RCT: randomized controlled trial, SD: standard deviation, PCI: percutaneous coronary intervention, and NR: not reported.

**Table 2:** Quality assessment of cohort studies using NOS.

Study Name	The Level of Representation of the Affected Cohort (★)	Identification of the Unexposed Cohort (★)	Determination of Exposure (★)	Evidence That the Outcome of Interest Was Absent at the Commencement of the Research (★)	Comparison of Cohorts Based on Design or Assessment (Max★★)	Was the Follow-Up Duration Sufficient for the Consequences to Manifest? (★)	Evaluation of Results (★)	Assessment of Cohort Follow-Up Sufficiency (★)	Quality Level
Ozasa et al., 2010 [29]	★	★	★	★	★★	★	★	★	High
López et al., 2020 [28]	★	★	★	★	★★	★	★	★	High
Al-Bawardy et al., 2024 [20]	★	-	★	★	★★	★	★	★	High
Choo et al., 2014 [27]	★	★	★	★	★★	-	★	★	High
Kernis et al., 2004 [26]	★	-	★	★	★★	★	★	★	High
Lee et al., 2015 [25]	★	★	★	★	★★	★	★	★	High
Joo et al., 2021 [24]	★	-	★	★	★★	★	★	★	High
Konishi et al., 2016 [23]	★	-	★	★	★★	-	★	★	High
Wen et al., 2022 [32]	★	★	★	★	★★	★	★	★	High
Bao et al., 2013 [22]	★	-	★	★	★★	★	★	★	High
Raposeiras-Roubin et al., 2015 [30]	★	-	★	★	★★	★	★	★	High
Yang et al., 2014 [21]	★	★	★	★	★★	★	★	★	High
Matsumoto et al., 2024 [31]	★	★	★	★	★★	★	★	★	High

## Data Availability

No new data were created or analyzed in this study.

## Supplementary Materials

The following supporting information can be downloaded at: https://www.mdpi.com/article/10.3390/jcm14113969/s1.
