# Supplementary material for: Exploring the Impact of Beta-Blockers Post-Acute Myocardial Infarction in Patients with Preserved Ejection Fraction: A Meta-Analysis
Source: J Clin Med. 2025 Jun 4;14(11):3969. doi: 10.3390/jcm14113969 (PMC12156146; doi:10.3390/jcm14113969)
Supplement: Supplementary file 1 [file jcm-14-03969-s001.zip › jcm-3527347-supplementary.pdf]

Study or Subgroup	Beta-blocker Events	Beta-blocker Total	No beta-blocker Events	No beta-blocker Total	Weight	Odds Ratio M-H, Fixed, 95% CI
Bao 2013	49	1137	49	1357	14.6%	1.20 [0.80, 1.80]
Joo et al 2021	109	6396	18	1230	10.2%	1.17 [0.71, 1.93]
Konishi 2016	1	197	7	227	2.2%	0.16 [0.02, 1.31]
Silvain 2024	44	1852	46	1846	15.4%	0.95 [0.63, 1.45]
Wen 2022	76	2042	20	470	10.7%	0.87 [0.53, 1.44]
Yang 2014	30	2650	19	1325	8.6%	0.79 [0.44, 1.40]
Yndigegn 2024	112	2508	117	2512	38.3%	0.96 [0.73, 1.25]
**Total (95% CI)**		**16782**		**8967**	**100.0%**	**0.97 [0.83, 1.14]**
Total events	421		276			

Heterogeneity: Chi² = 5.11, df = 6 (P = 0.53); I² = 0%
Test for overall effect: Z = 0.34 (P = 0.73)

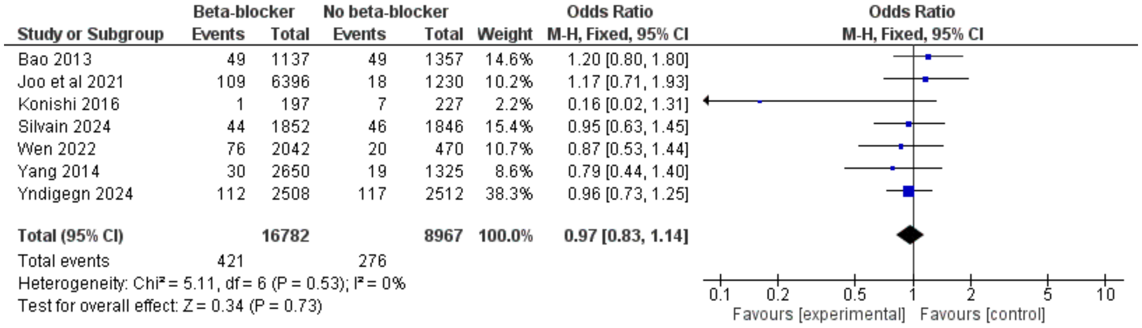

Supplementary Figure S1: The effect of beta-blockers on risk of recurrent MI using odds ratio

Study or Subgroup	log[Hazard Ratio]	SE	Weight	Hazard Ratio IV, Fixed, 95% CI
Bao 2013	0.174	0.2221	17.0%	1.19 [0.77, 1.84]
Choo 2014	-0.0305	0.3807	5.8%	0.97 [0.46, 2.05]
Joo et al 2021	0.207	0.2732	11.2%	1.23 [0.72, 2.10]
Wen 2022	-0.2231	0.2501	13.4%	0.80 [0.49, 1.31]
Yang 2014	-0.4943	0.3973	5.3%	0.61 [0.28, 1.33]
Yndigegn 2024	-0.0408	0.1328	47.4%	0.96 [0.74, 1.25]
**Total (95% CI)**			**100.0%**	**0.98 [0.82, 1.17]**

Heterogeneity: Chi² = 3.56, df = 5 (P = 0.61); I² = 0%
Test for overall effect: Z = 0.27 (P = 0.79)

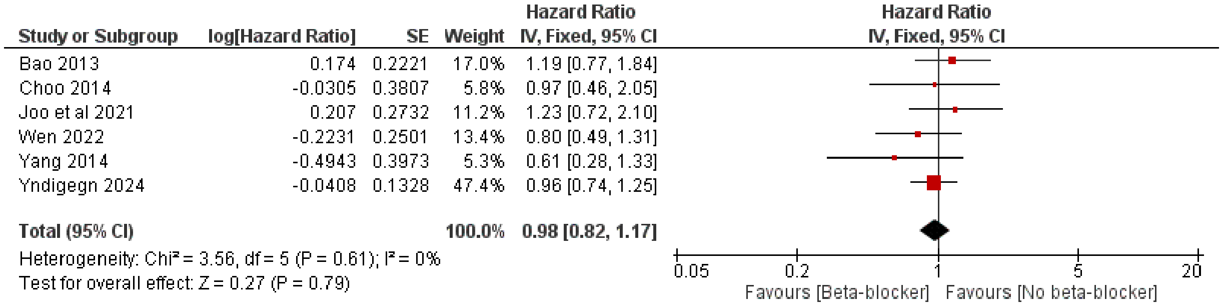

Supplementary Figure S2: The effect of beta-blockers on risk of recurrent MI using hazard ratio

Study or Subgroup	Experimental Events	Experimental Total	Control Events	Control Total	Weight	Odds Ratio M-H, Random, 95% CI
Bao 2013	54	1137	48	1357	12.1%	1.36 [0.91, 2.02]
Matsumoto 2024	1538	9905	537	3495	34.6%	1.01 [0.91, 1.13]
Silvain 2024	307	1852	349	1846	28.4%	0.85 [0.72, 1.01]
Wen 2022	718	2008	192	465	24.8%	0.79 [0.64, 0.97]
**Total (95% CI)**		**14902**		**7163**	**100.0%**	**0.94 [0.80, 1.11]**
Total events	2617		1126			

Heterogeneity: Tau² = 0.02; Chi² = 9.11, df = 3 (P = 0.03); I² = 67%
Test for overall effect: Z = 0.73 (P = 0.46)

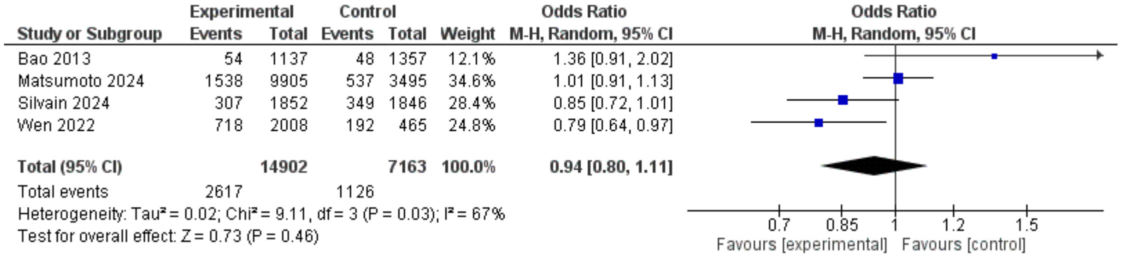

Supplementary Figure S3: The effect of beta-blockers on risk of rehospitalization using odds ratio

Study or Subgroup	log[Hazard Ratio]	SE	Weight	Hazard Ratio IV, Random, 95% CI
Matsumoto 2024	0.0677	0.0501	51.6%	1.07 [0.97, 1.18]
Wen 2022	-0.2744	0.0798	48.4%	0.76 [0.65, 0.89]
**Total (95% CI)**			**100.0%**	**0.91 [0.65, 1.27]**
Heterogeneity: Tau² = 0.05; Chi² = 13.18, df = 1 (P = 0.0003); I² = 92%				
Test for overall effect: Z = 0.57 (P = 0.57)				

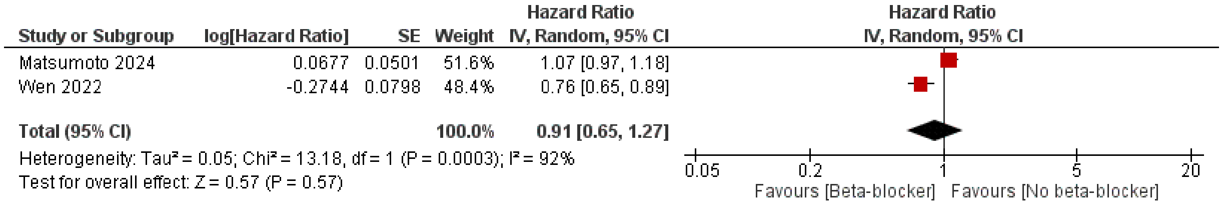

Supplementary Figure S4: The effect of beta-blockers on risk of rehospitalization using hazard ratio

Study or Subgroup	Beta-blocker Events	Beta-blocker Total	No beta-blocker Events	No beta-blocker Total	Weight	Odds Ratio M-H, Random, 95% CI
**1.12.1 RCTs**						
Silvain 2024	74	1852	76	1846	9.6%	0.97 [0.70, 1.34]
Watanabe 2018	20	394	24	400	5.6%	0.84 [0.46, 1.54]
Yndigegn 2024	97	2508	103	2512	10.3%	0.94 [0.71, 1.25]
**Subtotal (95% CI)**		**4754**		**4758**	**25.5%**	**0.94 [0.77, 1.15]**
Total events	191		203			
Heterogeneity: Tau² = 0.00; Chi² = 0.17, df = 2 (P = 0.92); I² = 0%						
Test for overall effect: Z = 0.60 (P = 0.55)						
**1.12.2 Cohort studies**						
Al-Bawardy 2024	215	6184	75	1148	10.5%	0.52 [0.39, 0.68]
Bao 2013	46	1137	71	1357	8.7%	0.76 [0.52, 1.12]
Joo et al 2021	162	6396	38	1230	9.0%	0.82 [0.57, 1.17]
Kernis 2004	36	1644	51	775	7.8%	0.32 [0.21, 0.49]
Lee 2015	8	137	26	137	3.7%	0.26 [0.12, 0.61]
Matsumoto 2024	1545	9905	667	3495	12.9%	0.78 [0.71, 0.87]
Ozasa 2010	13	217	11	350	3.8%	1.96 [0.86, 4.47]
Wen 2022	166	2049	39	470	9.0%	0.97 [0.68, 1.40]
Yang 2014	74	2650	54	1325	9.1%	0.68 [0.47, 0.97]
**Subtotal (95% CI)**		**30319**		**10287**	**74.5%**	**0.67 [0.53, 0.85]**
Total events	2265		1032			
Heterogeneity: Tau² = 0.09; Chi² = 36.55, df = 8 (P < 0.0001); I² = 78%						
Test for overall effect: Z = 3.25 (P = 0.001)						
**Total (95% CI)**		**35073**		**15045**	**100.0%**	**0.73 [0.61, 0.88]**
Total events	2456		1235			
Heterogeneity: Tau² = 0.07; Chi² = 41.72, df = 11 (P < 0.0001); I² = 74%						
Test for overall effect: Z = 3.26 (P = 0.001)						
Test for subgroup differences: Chi² = 4.38, df = 1 (P = 0.04), I² = 77.2%						

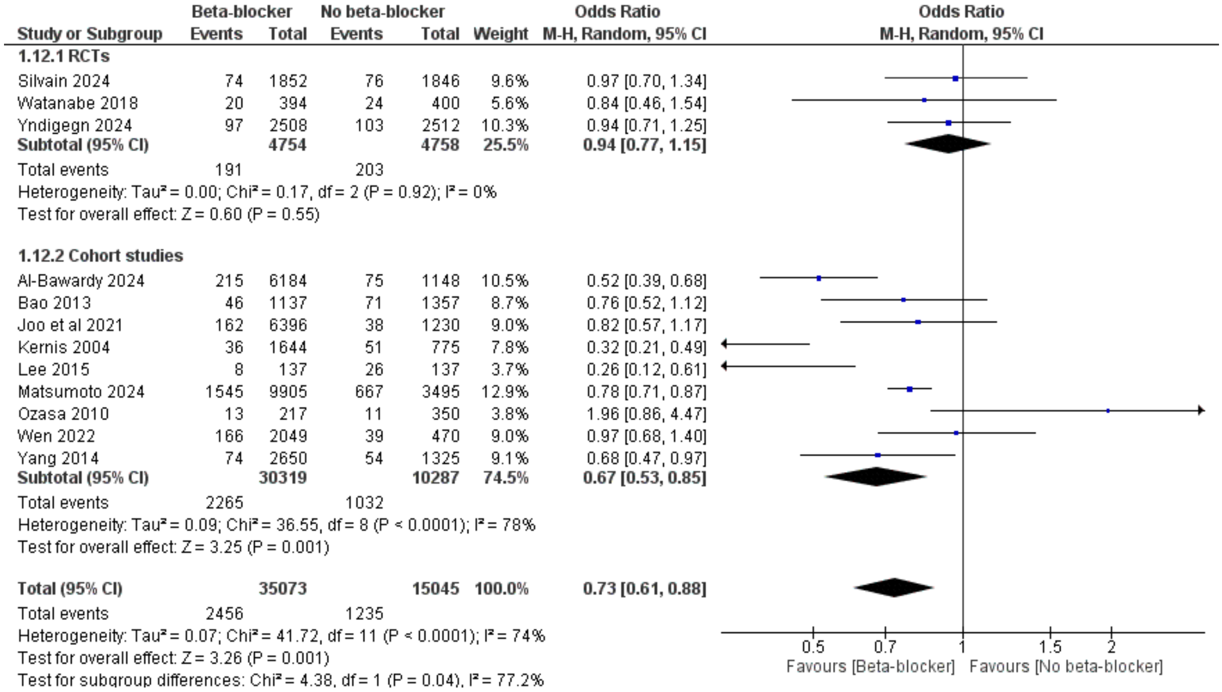

Supplementary Figure S5: Subgroup analysis of all-cause mortality outcome using odds ratio

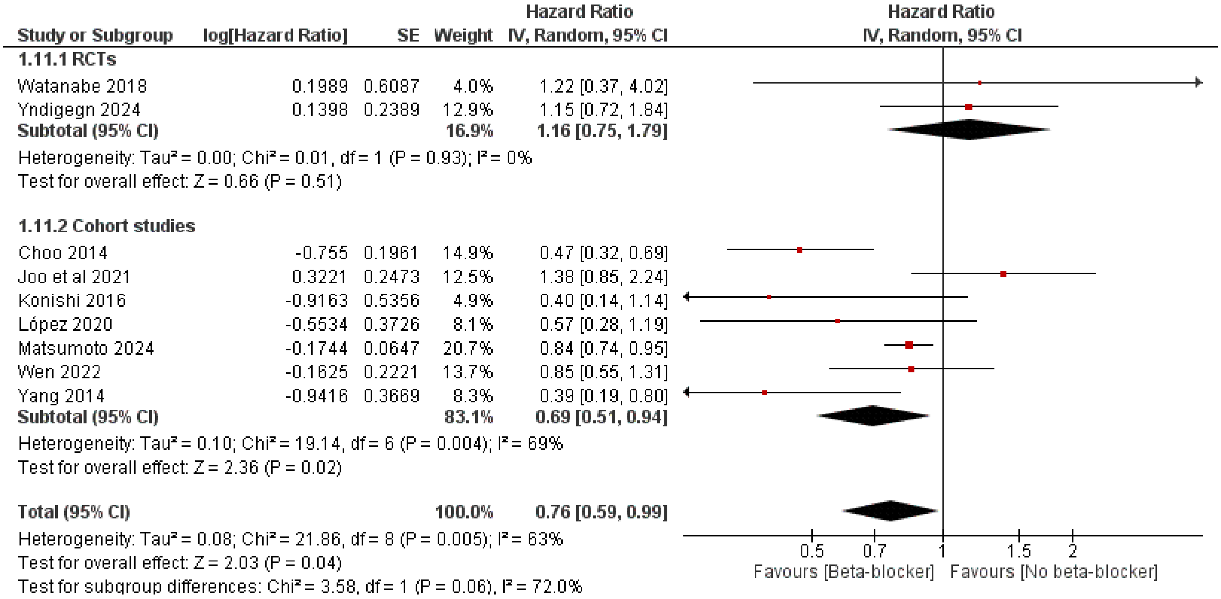

Supplementary Figure S6: Subgroup analysis of cardiac mortality outcome using hazard ratio

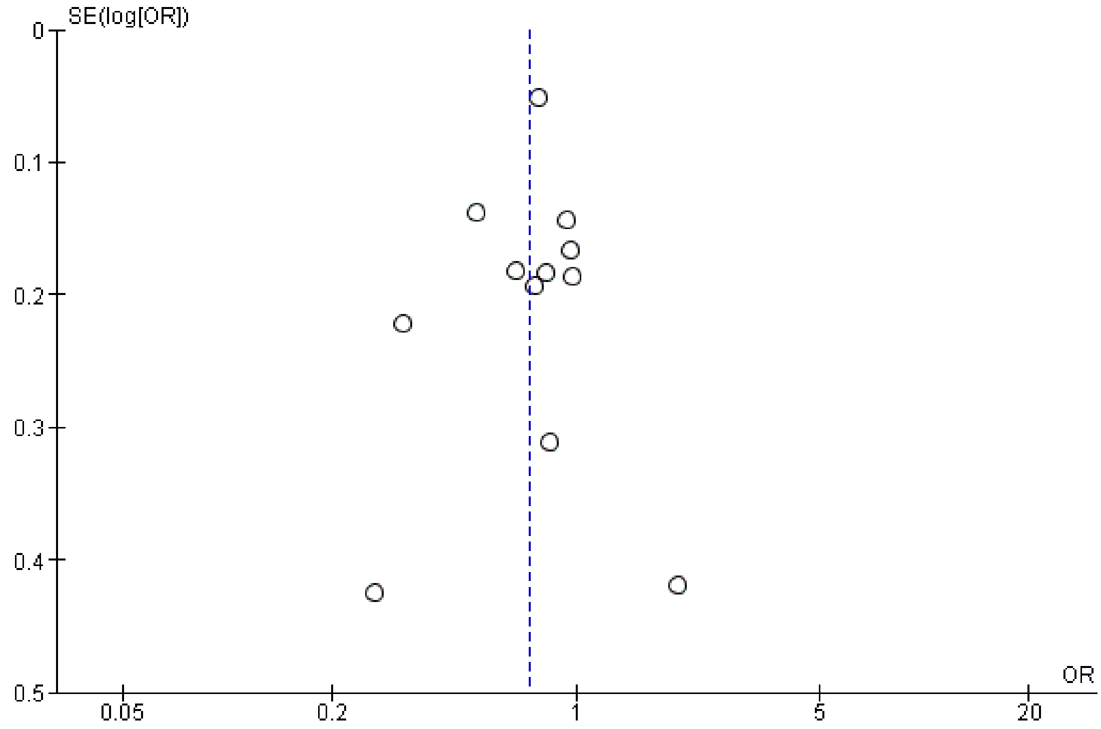

Supplementary Figure S7: Funnel plot for publication bias assessment of all-cause mortality using odds ratio

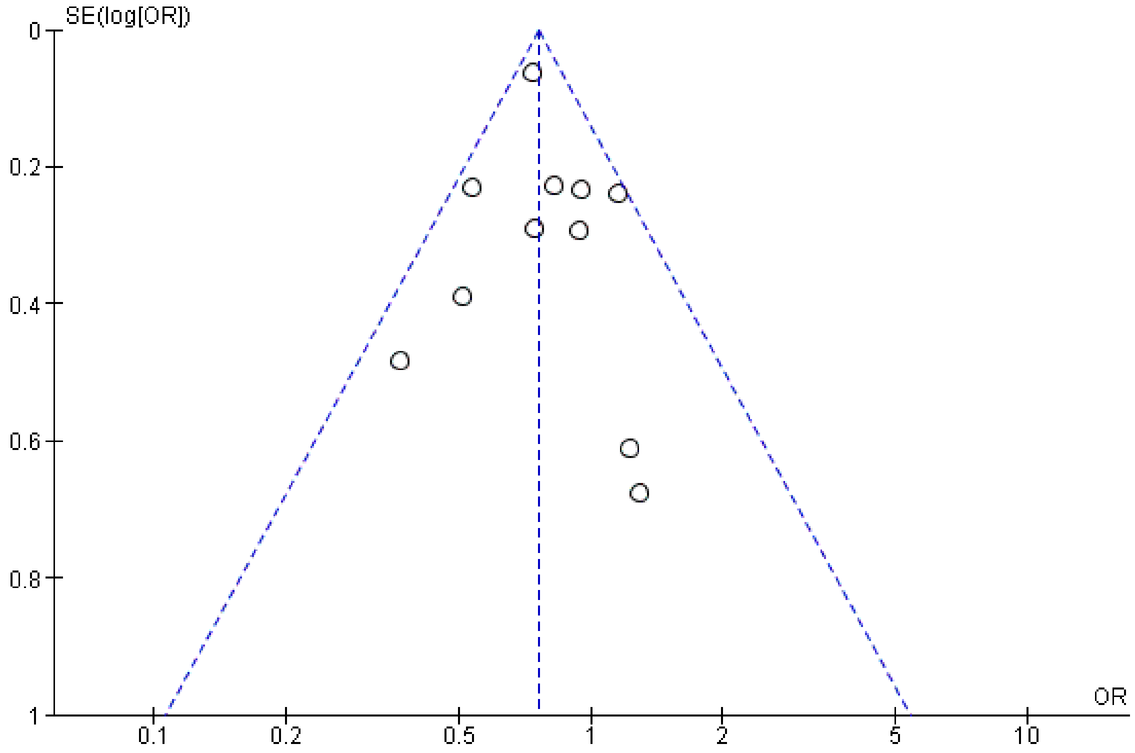

Supplementary Figure S8: Funnel plot for publication bias assessment of cardiac mortality using odds ratio

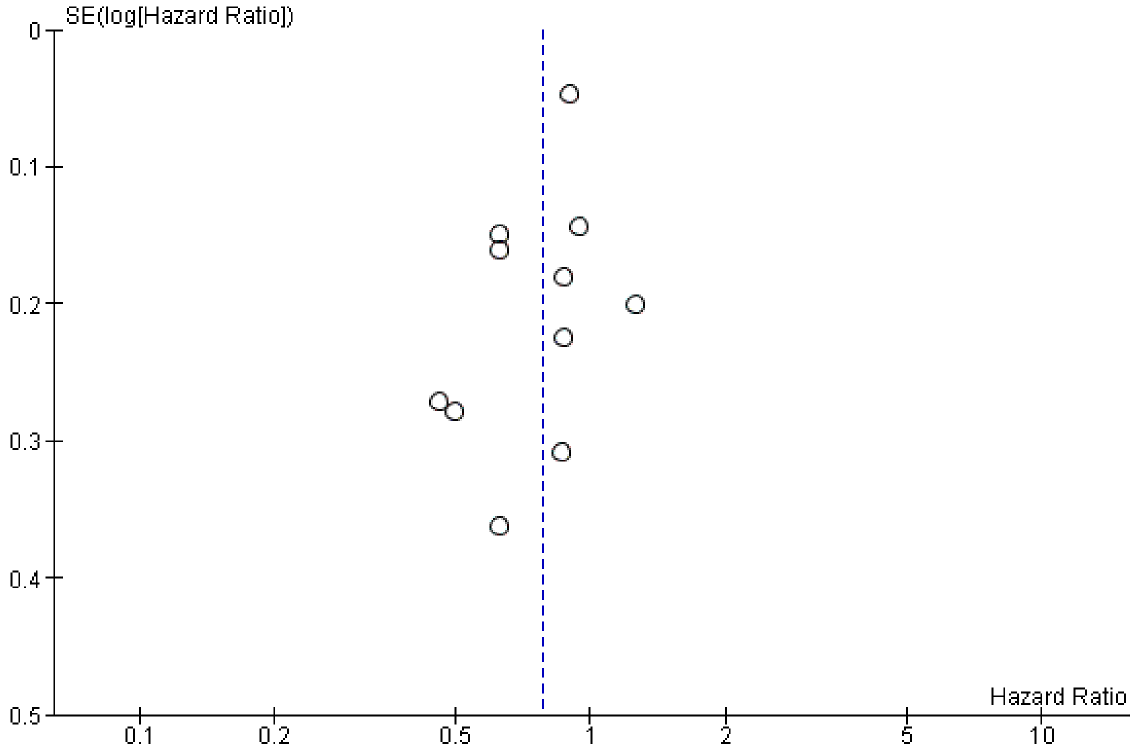

Supplementary Figure S9: Funnel plot for publication bias assessment of all-cause mortality using hazard ratio

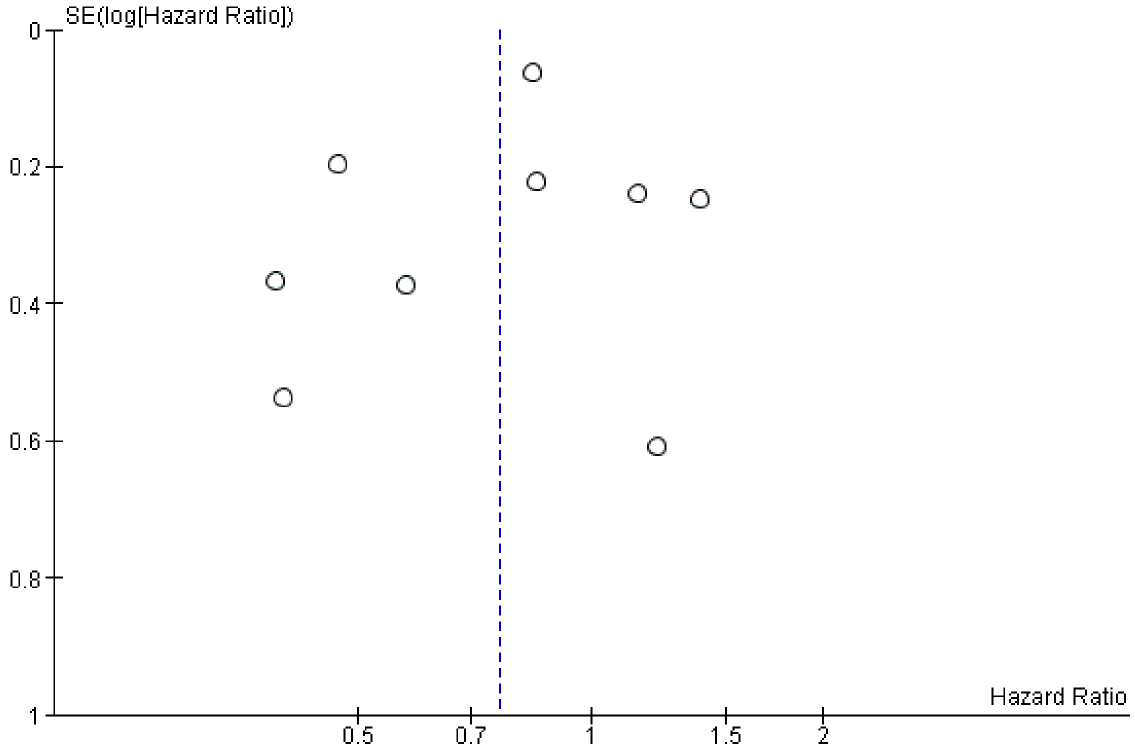

Supplementary Figure S10: Funnel plot for publication bias assessment of cardiac mortality using hazard ratio

Supplementary Table S1: Search strategies on different databases

Database	Search strategy
PubMed	((preserved[Title/Abstract]) AND (((((beta-Adrenoceptor Antagonists[Title/Abstract]) OR (beta-blockers[Title/Abstract])) OR (beta Adrenergic Antagonist[Title/Abstract])) OR (beta-Adrenergic Blocking Agent[Title/Abstract])) OR (beta Adrenergic Blocker[Title/Abstract]))) AND (((myocardial infarction[Title/Abstract]) OR (myocardial infarct[Title/Abstract])) OR (heart attack[Title/Abstract]))
Scopus	(ABS (preserved)) AND ((ABS (beta-blocker) OR ABS (beta-adrenoceptor AND antagonists) OR ABS (beta AND adrenergic AND antagonist) OR ABS (beta-adrenergic AND blocking AND agent) OR ABS (beta AND adrenergic AND blocker))) AND ((ABS (myocardial AND infarct) OR ABS (myocardial AND infarction) OR ABS (heart AND attack)))
Web of Science	(ABS (preserved)) AND ((ABS (beta-blocker) OR ABS (beta-adrenoceptor AND antagonists) OR ABS (beta AND adrenergic AND antagonist) OR ABS (beta-adrenergic AND blocking AND agent) OR ABS (beta AND adrenergic AND blocker))) AND ((ABS (myocardial AND infarct) OR ABS (myocardial AND infarction) OR ABS (heart AND attack)))
